# Hydrolysis of Arabinoxylo-oligosaccharides by α-L-Arabinofuranosidases and β-DXylosidase from *Bifidobacterium dentium*

**DOI:** 10.4014/jmb.2112.12021

**Published:** 2021-12-24

**Authors:** Min-Jae Lee, Yewon Kang, Byung Sam Son, Min-Jeong Kim, Tae Hyeon Park, Damee Park, Tae-Jip Kim

**Affiliations:** Division of Animal, Horticultural and Food Sciences, Graduate School of Chungbuk National University, Cheongju 28644, Republic of Korea

**Keywords:** *Bifidobacterium dentium*, α-L-arabinofuranosidases, β-D-xylosidase, arabinoxylo-oligosaccharides hydrolysis

## Abstract

Two α-L-arabinofuranosidases (BfdABF1 and BfdABF3) and a β-D-xylosidase (BfdXYL2) genes were cloned from *Bifidobacterium dentium* ATCC 27679, and functionally expressed in *E. coli* BL21(DE3). BfdABF1 showed the highest activity in 50 mM sodium acetate buffer at pH 5.0 and 25°C. This *exo*-enzyme could hydrolyze *p*-nitrophenyl arabinofuranoside, arabino-oligosaccharides (AOS), arabinoxylo-oligosaccharides (AXOS) such as 3^2^-α-L-arabinofuranosyl-xylobiose (A^3^X), and 2^3^-α-Larabinofuranosyl-xylotriose (A^2^XX), whereas hardly hydrolyzed polymeric substrates such as debranched arabinan and arabinoxylans. BfdABF1 is a typical *exo*-ABF with the higher specific activity on the oligomeric substrates than the polymers. It prefers to α-(1,2)-L-arabinofuranosidic linkages compared to α-(1,3)-linkages. Especially, BfdABF1 could slowly hydrolyze 2^3^,3^3^-di-α-L-arabinofuranosyl-xylotriose (A^2+3^XX). Meanwhile, BfdABF3 showed the highest activity in sodium acetate at pH 6.0 and 50°C, and it has the exclusively high activities on AXOS such as A^3^X and A^2^XX. BfdABF3 mainly catalyzes the removal of L-arabinose side chains from various AXOS. BfdXYL2 exhibited the highest activity in sodium citrate at pH 5.0 and 55°C, and it specifically hydrolyzed *p*-nitrophenyl xylopyranoside and xylo-oligosaccharides (XOS). Also, BfdXYL2 could slowly hydrolyze AOS and AXOS such as A^3^X. Based on the detailed hydrolytic modes of action of three *exo*-hydrolases (BfdABF1, BfdABF3, and BfdXYL2) from *Bf. dentium*, their probable roles in the hemiceulloseutilization system of *Bf. dentium* are proposed in the present study. These intracellular *exo*-hydrolases can synergistically produce L-arabinose and D-xylose from various AOS, XOS, and AXOS.

## Introduction

Hemicellulose polymers such as arabinans and xylans consist of the pentose sugars, L-arabinose and D-xylose, which are the low-calorie alternative sweeteners lowering blood glucose level by inhibiting the intestinal sucrase [1]. Recently, the prebiotic effects of arabino-, xylo-, and arabinoxylo-oligosaccharides (AOS, XOS, and AXOS) were intensively investigated on the probiotic microorganisms including *Lactobacillus* and *Bifidobacterium* species [2-4]. These hemicellulose-derived oligosaccharides selectively promote the growth of probiotic microorganisms to enhance the human gut health [5]. Arabinan polymers consist of α-(1,5)-L-arabinofuranosyl backbone and single- or double-substituted α-(1,2)-/α-(1,3)-L-arabinofuranosyl branches. *endo*-(1,5)-α-L-Arabinanases (ABNs, E.C. 3.2.1.99) can randomly hydrolyze the internal α-(1,5)-L-arabinofuranosidic linkages within arabinans to produce a series of AOS. Arabinoxylans are β-(1,4)-D-xylopyranosyl polymers with α-(1,2)-and/or α-(1,3)-L-arabinofuranosyl branches, which can be degraded into AXOS by *endo*-(1,4)-β-xylanases (XYNs, EC 3.2.1.8) [6]. For the enzymatic saccharification of the oligosaccharides into the fermentable sugars, the concerted actions of various *exo*-acting hydrolases are essential. α-L-Arabinofuranosidases (ABFs, EC 3.2.1.55) are the versatile *exo*-hydrolases which cleave α-(1,2)-, α-(1,3)-, and/or α-(1,5)-L-arabinofuranosidic linkages of AOS and α-(1,2)- and/or α-(1,3)-L-arabinofuranosyl branches of AXOS to produce only L-arabinose as an end-product [7, 8]. Meanwhile, β-D-xylosidases (XYLs, E.C. 3.2.1.37) are the key players hydrolyzing β-1,4-D-xylopyranosidic linkages of XOS to liberate D-xylose exclusively [8].

In the human gastrointestinal tract, the genus *Bifidobacterium* is known as one of the major probiotic bacteria with the health-promoting properties [9]. The selective prebiotic effects of hemicellulose-derived oligosaccharides on *Bifidobacterium* spp. are known to be structure-dependent and strain-specific [10]. For the efficient utilization of prebiotic oligosaccharides, the target probiotic microorganisms should possess the sets of corresponding *exo*- and *endo*-hydrolase genes, and their expression can be properly regulated [11, 12]. Recent genomic, transcriptomic, and proteomic approaches revealed that *Bifidobacterium* spp. sensitive to the prebiotic AXOS can timely express any combination of core arabinoxylan-degrading enzyme genes including ABF and XYL [13, 14]. To date, several ABFs have been studied from *Bf. adolescentis* [15, 16], *Bf. longum* [17-19], and *Bf. breve* [20]. Also, a few XYLs has been reported from *Bf. adolescentis* [21], *Bf. longum*, and *Bf. breve* [22]. Although *exo*-hydrolases were previously cloned from some *Bifidobacterium* species, their enzymatic characterizations were mainly carried out with a few simple-structured substrates such as *p*-nitrophenyl sugars. Therefore, the detailed hydrolytic modes of action and preferences towards various natural complex substrates have not been well-understood to date. Unlike most common Bifidobactrium species being frequently isolated from the gastrointestinal tract, *Bf. dentium* spp. are identified from the oral cavity, dental caries, or feces of human and animals [23]. Although *Bf. dentium* is recently considered as one of major *Bifidobacterium* species, the information about its enzymatic hemicellulose-utilization system and related genes are not focused yet. Based on the microbial genome information, meanwhile, five open reading frames probably encoding *exo*-acting hemicellulose-hydrolases were found *Bf. dentium* ATCC 27679 which was isolated from a human vagina.

In this study, the genes encoding two ABFs (hereafter, BfdABF1 and BfdABF3) and a XYL (BfdXYL2) were cloned from *Bf. dentium*, and their enzymatic properties, substrate specificities, and hydrolytic modes of action towards various natural substrates such as AXOS were comparatively characterized in detail. Finally, the roles of these *exo*-hydrolases were proposed for the degradation and utilization of AXOS in *Bf. dentium*.

## Materials and Methods

### Enzyme Substrates

Various carbohydrate substrates were supplied by Megazyme (Ireland). The abbreviations of polymeric and oligomeric substrates were summarized as follow: xylo-, arabino-, and arabinoxylo-oligosaccharides (XOS, AOS, and AXOS), rye and wheat arabinoxylans (RAX and WAX), beechwood xylan (BEX), sugar beet arabinan (SA), debranched arabinan (DA), X1~X6 (D-xylose to xylohexaose), A1~A7 (L-arabinose to arabinoheptaose), A^3^X (3^2^-α-L-arabinofuranosyl-xylobiose), A^2^XX (2^3^-α-L-arabinofuranosyl-xylotriose), A^3^XX (3^3^-α-L-arabinofuranosyl-xylotriose), A^2+3^XX (2^3^,3^3^-di-α-L-arabinofuranosyl-xylotriose), XA^2^XX (2^3^-α-L-arabinofuranosyl-xylotetraose), XA^3^XX (3^3^-α-L-arabinofuranosyl-xylotetraose), and XA^2+3^XX (2^3^,3^3^-di-α-L-arabinofuranosyl-xylotetraose). The synthetic substrates, *p*-nitrophenyl-α-L-arabinofuranoside and *p*-nitrophenyl-β-D-xylopyranoside (*p*-NPAf and *p*-NPXp), were purchased from Sigma-Aldrich (USA).

### Gene Cloning, Expression, and Enzyme Purification

*Bifidobacterium dentium* ATCC 27679 was grown in Tryptic soy broth with 5% defibrinated sheep blood at 37°C, and genomic DNA template was isolated by using DNeasy Blood & Tissue kit (Qiagen, Germany). Five pairs of PCR primers, BfdABF1-N (5’-TTTTCATATGGACAAGCTCACCG-3’) and BfdABF1-C (5’-TTTTCT CGAGTTTGATGGTGATGATGCT-3’), BfdABF2-N (5’-TTTTCATATGACCATCCAAGATTC-3’) and BfdABF2-C (5’-TTTTCTCGAGGCAAACATCTCCTTCC-3’), BfdABF3-N (5’-TTTTGCTAGCATGACGATTGCAG CAACAA-3’) and BfdABF3-C (5’-TTTTAAGCTTATCGGACAATCCCGCCAT-3’), Bfd XYL1-N (5’-TTTTCA TATGCTGCACAATCCGA-3’) and BfdXYL1-C (5’-TTTTCTGCAG GTCGACCGGCTTGG-3’), and BfdXYL2-N (5’-TTTTCATATGCAGATTTCCAACCC-3’) and BfdXYL2-C (5’-TTTTCTCGAGTTCGCCGTCAGGAAGT-3’), were designed to amplify the target genes encoding BfdABF1 (GenBank ID: EFM40505.1), BfdABF2 (EFM42519.1), BfdABF3 (EFM41056.1), BfdXYL1 (EFM42357.1), and BfdXYL2 (EFM41051.1), respectively. Gene amplification was performed by using Pyrobest polymerase (Takara, Japan) and thermal cycler C-1000 (Bio-Rad, UK) with the following steps: an initial denaturation at 98°C for 30 sec followed by 30 repeated cycles at 98°C for 10 sec, 54°C for 30 sec, 72°C for 1 min 30 sec, and an additional polymerization at 72°C for 5 min. The amplified DNA fragments were cleaved with NdeI/XhoI (for BfdABF1, ABF2, and XYL2), NheI/HindIII (BfdABF3), NdeI/PstI (BfdXYL1) and cloned into the IPTG-inducible expression vector, pET-21a (Novagen, Germany). The resulting recombinant plasmid was designated as pETBfdABF1, pETBfdABF3, and pETBfdXYL2, respectively. For the gene expression, *Escherichia coli* BL21(DE3) transformant was cultured in LB broth with 0.1 mM IPTG and 100 μg/ml ampicillin at 37°C for 15 h. The grown cells were disrupted by ultrasonicator VCX750 (Sonics & Materials, USA). Each enzyme fused with six-histidines at C-terminus was purified via HisTrap FF column chromatography by using AKTA Prime (GE Healthcare, Sweden). The level of gene expression and the enzyme purity was analyzed by using 12% SDS-PAGE. The protein concentration was measured by BCA protein assay kit (Pierce Biotechnology, USA).

### Enzyme Activity Assays

The enzyme activity on 1 mM *p*-nitrophenyl sugars (*p*-NPAf and *p*-NPXp) was determined at 405 nm by measuring the *p*-nitrophenol being released. L-Arabinose/D-Galactose assay kit (Megazyme) was utilized to measure L-arabinose produced from 0.5% polymeric or oligomeric substrates at 340 nm. One unit of ABF or XYL activity was defined as the amount of enzyme liberating 1 μmol of L-arabinose, D-xylose, or *p*-nitrophenol from each substrate for 1 min, respectively.

### Thin Layer Chromatography (TLC) Analysis

The enzymatic hydrolysis patterns were comparatively analyzed by using thin layer chromatography (TLC). At the optimal condition, 0.5% of substrate was reacted with ABF or XYL for an appropriate time, and the resulting hydrolysates were separated on a 60F_254_ silica gel glass TLC plate (Merck, Germany) with the solvent of chloroform/acetate/water (6:7:1). The product spots were visualized and identified by the developing solution (0.3% N-1-naphthyl-ethylenediamine and 5% H_2_SO_4_ in methanol) at 110°C for 10 min.

### High performance Anion Exchange Chromatography (HPAEC) Analysis

Bio-LC system (ICS-3000; Thermo-Fisher, USA) equipped with a CarboPac PA1 column (4 × 250 mm) and an electrochemical detector (ED40) was utilized for the analysis of enzymatic hydrolysates. The samples were eluted with a linear gradient from 100% 150 mM NaOH (buffer A) to 15% buffer B (600 mM sodium acetate in buffer A) over 40 min. The flow rate of the mobile phase maintained at 1.0 ml/min through the analysis.

## Results and Discussion

### Gene Cloning and Expression of BfdABFs and BfdXYLs

**Fig. 1:**
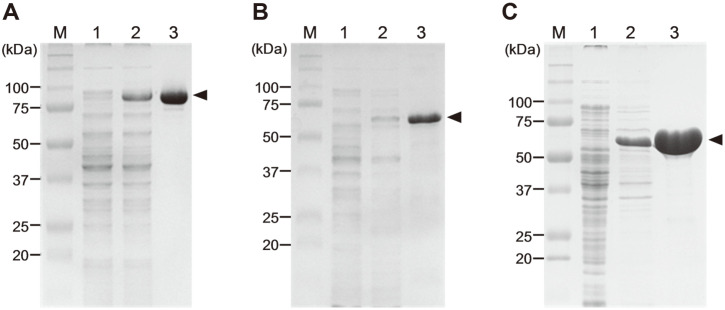
Gene expression and enzyme purification of (**A**) BfdABF1, (**B**) BfdABF3, and (**C**) BfdXYL2 with a Cterminal 6xHis-tag from *E. coli*. The expression level and enzyme purity were examined using SDS-PAGE analysis. Lane M, protein size marker; 1, cell extract from *E. coli* with an empty vector (negative control); 2, cell extract from *E. coli* harboring pETBfdABF or pETBfXYL; 3, recombinant enzyme purified by Ni-NTA chromatography (arrowheads).

Probable genes encoding three α-L-arabinofuranosidases (BfdABF1, 2, and 3) and two β-D-xylosidases (BfdXYL1 and 2) were found in the genome of *Bf. dentium* ATCC 27679, and cloned into an IPTG-inducible expression vector, pET-21a. Of these five genes, BfdABF2 and BfdXYL1 did not exhibit the detectable levels of gene expression and enzymatic activity on any of the substrates tested in this study. In contrast, the three genes encoding C-terminal 6-histidines-tagged BfdABF1, BfdABF3, and BfdXYL2 were successfully expressed in *E. coli* BL21(DE3), and purified by an Ni-NTA affinity chromatography (Fig. 1). The open reading frames of BfdABF1, BfdABF3, and BfdXYL2 encode 773 (85,395 Da), 572 (65,372 Da), and 543 (59,011 Da) amino acids, respectively. The SignalP 5.0 analysis predicted that all these *exo*-hydrolases without a detectable signal peptide sequence would likely be expressed as the intracellular enzymes. The apparent molecular mass of recombinant BfdABF1 (85 kDa), BfdABF3 (65 kDa), and BfdXYL2 (59 kDa) were similar to those being expected from the deduced amino acid sequences.

### Optimal Reaction Conditions for BfdABFs and BfdXYL

**Fig. 2:**
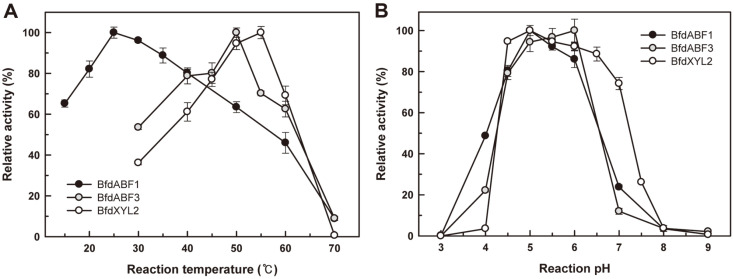
Effects of (**A**) temperature and (**B**) pH on enzyme activities of BfdABF1, BfdABF3, and BfdXYL2. Relative activities of ABF and XYL on *p*-NPAf and *p*-NPXp were respectively measured at 405 nm. Sodium citrate (pH 3~6), sodium acetate (pH 4~6), sodium phosphate (pH 6~8), and Tris-HCl (pH 7~9) buffers were used for the activity assay.

BfdABF1 showed the highest activity on *p*-NPAf in 50 mM sodium acetate buffer at pH 5.0 and 25°C (Fig. 2). Its enzymatic activity at pH 4.0 and 7.0 was less than 50% of optimal conditions. In contrast, BfdABF3 was highly active in 50 mM sodium citrate buffer at pH 5.0~6.0 and sodium acetate buffer at pH 5.5~6.0 at 50°C. Most known microbial ABFs have their own optimal reaction conditions at pH 5.0~7.0 and 40~60°C [7]. Similarly, it was known that the ABFs from lactic acid bacteria show the highest activity at pH 5.5~6.0 and 30~50°C. For example, AbfA and AbfB from *Bf. adolescentis* ATCC 15703 exhibited the highest activity at pH 6.0, and 30°C and 50°C, respectively [16]. The ABF from *Levilactobacillus brevis* DSM 20054 has an optimal temperature at 60~62°C and pH 5.0~5.5 [24].

BfdXYL2 showed the highest activity in 50 mM sodium acetate buffer at pH 5.0 and 55°C (Fig. 2). It was highly active in 50 mM sodium phosphate buffer at pH 6.0~6.5 as well. In the case of *Bf. adolescentis* LMG 10502, two β-xylosidases (XylB and XylC) showed the highest activities at pH 5.5 and 60°C, and pH 6.0 and 50°C, respectively [21]. A β-xylosidase (BXA43) from *Bf. animalis* subsp. *lactis* BB-12 has the optimum of pH 5.5 and 50°C [25], and XynB2 from Le. brevis DSM 20054 showed the highest activity at pH 6.0 and 50°C [26].

### Substrate Specificity of BfdABFs and BfdXYL

The ABF activity against various substrates was measured using *p*-nitrophenyl sugar assay, DNS reducing sugar assay, and L-arabinose assay kit (Table 1). BfdABF1 has very weak, but detectable activity (0.23 U/mg) against branched SA polymer, whereas the hydrolytic activity against linear DA and various xylans was not significant. In contrast, BfdABF1 showed significantly high activity on oligomeric substrates, such as *p*-NPAf, AOS, and AXOS. These results revealed that BfdABF1 is an *exo*-type ABF specific to AOS and AXOS, not polymeric substrates. Among the various AOS, arabinobiose is the most preferred substrate for BfdABF1. Its hydrolytic activity against other AOS is less than 64% of that against arabinobiose. For AXOS, BfdABF1 could hydrolyze both A^3^X and A^2^XX. Its activity on A^2^XX was about 70% of that on A^3^X, while the activities on XA^3^XX and A^2+3^XX were very low. This means that BfdABF1 possesses high activity removing the single-substituted α-(1,2)- or α-(1,3)-arabinofuranosyl residues linked to D-xylose at the non-reducing terminus, not those linked to the internal D-xylose of XOS. Double-substituted AXOS, such as A^2+3^XX and XA^2+3^XX, were very slowly hydrolyzed when the excess amount of BfdABF1 was treated.

BfdABF3 has no detectable activity against most arabinan and xylan polymers, AOS, as well as *p*-NPAf (Table 1). In contrast, BfdABF3 has the highest activity on A^2^XX (71.4 U/mg), and considerable levels of activity on A^3^X (5.57 U/mg) and XA^3^XX (1.43 U/mg). On the contrary, the double-substituted AXOS including A^2+3^XX and XA^2+3^XX could not be hydrolyzed by BfdABF3. These results suggest that BfdABF3 is a single-substituted AXOS-specific *exo*-ABF. This enzyme can be easily distinguished from BfdABF1 due to the lack of detectable activity for AOS and *p*-NPAf, and a 16-fold higher and more specific debranching activity for A^2^XX.

BfdXYL2 showed very low activity (0.23 U/mg) only on beechwood xylan, but no activity on wheat and rye arabinoxylans, and arabinan polymers. The hydrolytic activity of BfdXYL2 on *p*-NPXp (12.14 U/mg) was much higher than that on *p*-NPAf (0.53 U/mg). As expected, BfdXYL2 was highly active against most linear XOS substrates. Therefore, BfdXYL2 is considered as an *exo*-type β-D-xylosidase highly specific for the β-D-xylopyranosidic linkages of linear XOS, not for AXOS and arabinoxylan polymers. Among XOS, BfdXYL2 showed the highest activity against the shortest xylobiose, and less than 41% of activity on xylotriose and the longer XOS. Although BfdXYL2 had much lower activity, it had the detectable activity against arabinobiose (0.13 U/mg) and A^3^X (0.17 U/mg), respectively, whereas its activities against A^2^XX, A^2+3^XX, and XA^2+3^XX were marginal.

### Hydrolytic Modes of Action of BfdABFs and BfdXYL

**Fig. 3:**
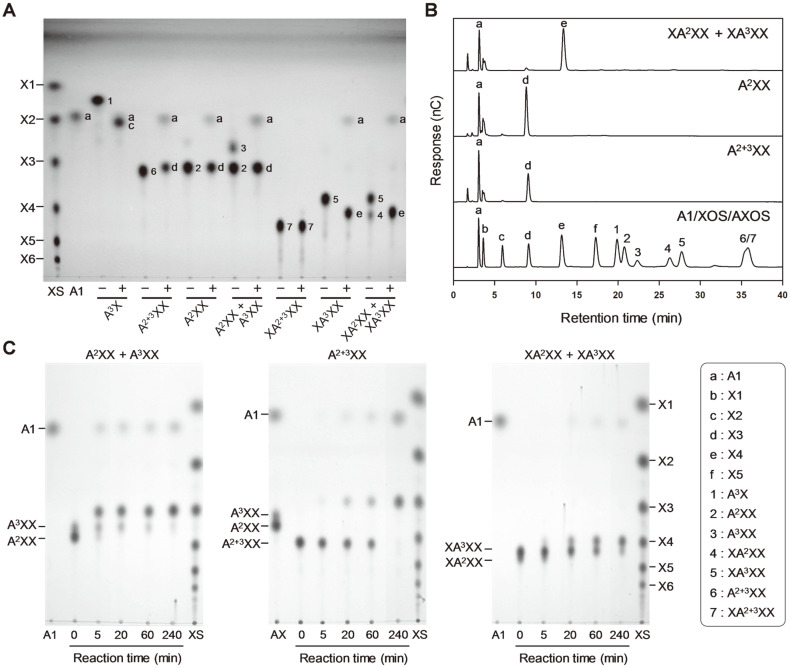
AXOS hydrolysis patterns of BfdABF1 analyzed using (**A**) TLC analysis, (**B**) HPAEC analysis (**C**) timecourse analysis. For the end-point analysis, BfdABF1 (1 U/ml) was reacted with 0.5% of each substrate under the optimal condition for 12 h, whereas for the time-course analysis, 0.01 U/ml was used. (-) and (+) indicate the samples reacted without and with enzyme, respectively. A1, L-arabinose standard; XS, XOS standard. Abbreviations for various oligosaccharides are shown in the figure.

To investigate the detailed hydrolytic actions towards AXOS, XOS, and AOS, three *exo*-hydrolases were respectively reacted with various substrates, and the reaction products were comparatively analyzed with each other using TLC and HPAEC methods. After the excess amount of enzyme (1 U/ml) was reacted with AXOS for 15 h, BfdABF1 could cleave L-arabinose residues from A^3^X, A^2+3^XX, A^2^XX, A^3^XX, XA^2^XX, and XA^3^XX, while it had no activity on XA^2+3^XX (Figs. 3A and 3B). Time-course TLC analysis showed that BfdABF1 hydrolyze both α-(1,2)- and α-(1,3)-L-arabinofuranosyl linkages from the mixture of A^2^XX and A^3^XX to L-arabinose and xylotriose (Fig. 3C). It was observed that the hydrolytic rate of A^2^XX is relatively faster than that of A^3^XX. Even though BfdABF1 very slowly hydrolyzed A^2+3^XX, this enzyme preferentially attacked α-(1,3)-L-arabinofuranosidic linkages. At the initial reaction step, BfdABF1 slowly hydrolyzed α-(1,3)-linkage of A^2+3^XX to produce L-arabinose and A^2^XX, and then the resulting A^2^XX was rapidly degraded into arabinose and xylotriose as the final products. Accordingly, the intermediate A^2^XX or A^3^XX was not observed at all. Similarly, BfdABF1 could slowly hydrolyze the mixture of XA^2^XX and XA^3^XX to generate L-arabinose and xylotetraose. As XA^2^XX was rapidly disappeared, the amounts of L-arabinose and xylotetraose proportionally increased. Although XA^2^XX contains the internal α-(1,2)-L-arabinofuranosidic side chain, its hydrolysis was much faster than that of XA^3^XX. In contrast, the hydrolysis of XA^3^XX was too slow to be detected within 30 min, which coincides with the extremely low activity of BfdABF1 on XA^3^XX (Table 1).

**Fig. 4:**
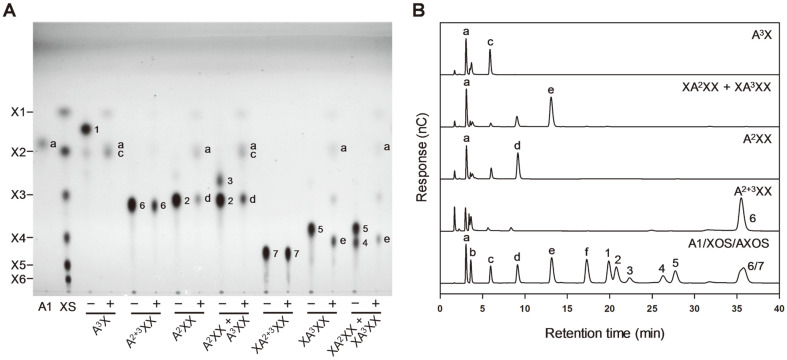
AXOS hydrolysis patterns of BfdABF3 analyzed using (**A**) TLC analysis and (**B**) HPAEC analysis. BfdABF3 (1 U/ml) was reacted with 0.5% of each substrate under the optimal condition for 12 h. (-) and (+) indicate the samples reacted without and with enzyme, respectively. A1, L-arabinose standard; XS, XOS standard. Abbreviations for various oligosaccharides are listed in Fig. 3.

BfdABF3 shares very similar AXOS hydrolysis patterns with BfdABF1, with the exception of A^2+3^XX (Fig. 4). BfdABF3 could not hydrolyze A^2+3^XX as well as XA^2+3^XX, whereas BfdABF1 hydrolyzed A^2+3^XX to liberate L-arabinose residues. As expected from remarkably high specific activity of BfdABF3 against A^2^XX in Table 1, the time-course TLC analysis also showed that the hydrolysis of A^2^XX or XA^2^XX by BfdABF3 is much faster than that of A^3^XX or XA^3^XX (data not shown).

**Fig. 5:**
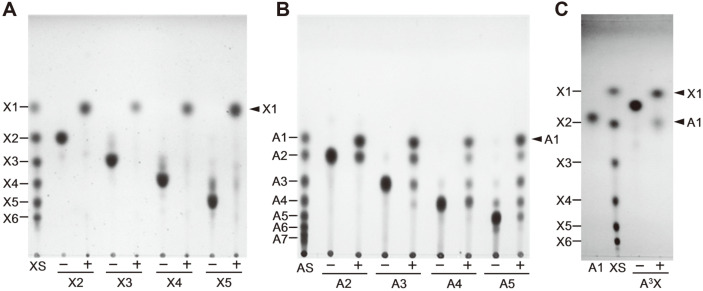
Hydrolysis patterns of BfdXYL2 on (**A**) XOS and (**B**) AOS (**C**) A^3^X analyzed using TLC. BfdXYL2 (1 U/ml) was reacted with 0.5% of each substrate under the optimal condition for 12 h. (-) and (+) indicate the samples reacted without and with enzyme, respectively. XS, XOS standard; AS, AOS standard; X1, D-xylose; A1, L-arabinose.

According to TLC analysis, BfdXYL2 could cleave mainly β-(1,4)-D-xylopyranosidic linkages of XOS substrates. As shown in Table 1 and Fig. 5A, BfdXYL2 is a typical *exo*-hydrolase exhibiting much higher activity against xylobiose, the shortest substrate, than the longer oligomeric and polymeric substrates. Even though the activity of BfdXYL2 against AOS was much lower than that against XOS, this enzyme could very slowly and partly remove the L-arabinose residues from the non-reducing end of AOS (Fig. 5B). A series of AOS intermediates was observed from the incomplete hydrolysis of AOS by BfdXYL2 because of the much lower activity against AOS. Especially, BfdXYL2 could hydrolyze A^3^X into L-arabinose and D-xylose (Fig. 5C). This result implies that BfdXYL2 slowly but distinctly cleaved α-(1,3)-L-arabinofuranosyl linkage of A^3^X, and the resulting xylobiose was rapidly hydrolyzed into D-xyloses.

*Bf. adolescentis* LMG10502 (or ATCC 15703) was reported to produce three different ABFs [16]. Among them, AbfA and AbfB share approximately 80 and 62% of amino acid sequence identities with those of BfdABF1 and ABF3, respectively. *Bf. adolescentis* AbfA is an *exo*-hydrolase belonging to GH43 family which degrades α-(1,2)- or α-(1,3)-L-arabinofuranosyl linkages from single-substituted AXOS, while AbfB GH51 selectively hydrolyzes only α-(1,3)-L-arabinofuranosyl linkages from double-substituted AXOS. BfdABF1 shares similar hydrolytic activity and substrate specificity with those of *Bf. adolescentis* AbfA, whereas BfdABF3 exhibits largely different hydrolytic modes of action from AbfB [16] and AXH-d3 [15]. BfdXYL2 is closely similar to *Bf. adolescentis* β-xylosidase GH43 (XylC) which prefers to XOS including xylobiose [21]. Although the hydrolytic activity of BfdXYL2 against AOS was much low, this hydrolase showed the significant activities against both XOS and AOS. A XOS-upregulated bifunctional XYL/ABF GH43 (BXA43) was reported from the probiotic *Bf. animalis* subsp. *lactis* BB-12, which shares about 76% amino acid sequence identity with that of BfdXYL2 [25]. It was reported that a multi-functional glycoside hydrolase (Blon_0625) from *Bf. longum* subsp. *infantis* possesses the considerable activities towards *p*-nitrophenyl-β-D-glucoside as well as *p*-NPXp and *p*-NPAf [27]. Recently, AXOS- and AOS-specific ABFs GH51 were functionally characterized from an amylolytic yeast, *Saccharomycopsis fibuligera* [28]. Although these ABFs similarly hydrolyzed only oligomeric substrates, not polymeric substrates, they showed no significant amino acid identities with BfdABF1 and 3.

### Proposed Roles of *exo*-Hydrolases in *Bf. dentium*

**Fig. 6:**
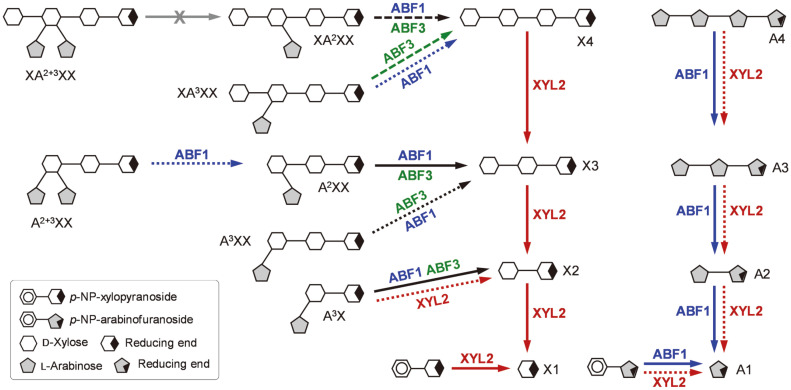
Hydrolytic modes of action of BfdABF1, BfdABF3, and BfdXYL2 on various AXOS and AOS. The solid, long-dashed, and short-dashed arrows indicate the high, intermediate, and low hydrolyzing activities of BfdABFs and BfdXYL against each substrate, respectively.

The detailed hydrolytic modes of action of BfdABF1, ABF3, and XYL2 on various oligosaccharide substrates have been schematically summarized (Fig. 6). In conclusion, BfdABF1 is a typical *exo*-type α-L-arabinofuranosidase that removes only L-arabinose by acting on various AOS and branched AXOS. BfdABF3 is supposed to be a debranching enzyme which does not act on most common substrates for ABF including *p*-NPAf and AOS, but possesses the considerable activity only towards various AXOS substrates, including A^2^XX and A^3^X. On the other hand, BfdXYL2 is a typical β-D-xylosidase specific for β-(1,4)-D-xylopyranosidic linkages of XOS and beechwood xylan.

Recently, to help the understanding of the synbiotics (probiotics and prebiotics) system, the relationship between the synergistic action of microbial carbohydrate-activating enzymes and the target substrate was intensively studied [12, 13]. In this study, two ABF and XYL genes were functionally expressed from *Bf. dentium* and their enzymatic properties were comparatively characterized with each other. Based on the hydrolysis pattern analysis, it was predicted that three *exo*-type hydrolases can synergistically hydrolyze AXOS to produce the fermentable monosaccharides, L-arabinose and D-xylose. By trimming the L-arabinosyl branches by BfdABF1 and ABF3, for instance, AXOS are converted to the linear XOS, which can be further decomposed into D-xylose by the successive actions of BfdXYL2. The resulting pentose sugars are expected to act as the prebiotics which can promote the growth of *Bifidobacterium* species [2, 3]. For the more efficient arabinoxylan-utilization by *Bf. dentium*, however, the *endo*-type β-xylanases and the *exo*-type ABFs will be additionally necessary. β-Xylanases shorten arabinoxylan polymers to produce AXOS, which can be further degraded into L-arabinose and D-xylose by ABFs with the specific activities towards the double-substituted and/or α-(1,3)-L-arabinofuranosyl branches of AXOS.

## Figures and Tables

**Table 1:** Specific activities of BfdABFs and BfdXYL on various hemicellulosic substrates.

Type	Substrate	Specific activity (U/mg)^a^		

		BfdABF1	BfdABF3	BfdXYL2
Synthetic	*p*-NPAf	5.53±0.13	ND^b^	0.53±0.04
	*p*-NPXp	ND	ND	12.14±1.03
AOS	A2	3.84±0.43	ND	0.13±0.02
	A3	2.46±0.15	ND	0.05±0.01
	A4	2.09±0.20	ND	0.04±0.01
	A5	2.14±0.06	ND	0.03±0.00
	A6	2.23±0.12	ND	0.04±0.01
XOS	X2	ND	ND	4.43±0.23
	X3	ND	ND	1.51±0.22
	X4	ND	ND	1.83±0.11
	X5	ND	ND	1.56±0.07
	X6	ND	ND	1.33±0.01
AXOS	A^3^X	6.52±0.11	5.57±0.25	0.17±0.03
	A^2^XX	4.54±0.58	71.40±1.12	ND
	XA^3^XX	0.12±0.02	1.43±0.10	ND
	A^2+3^XX	0.07±0.01	ND	ND
Polymer	SA	0.23±0.04	ND	ND
	BEX	ND	ND	0.23±0.03

^a^Enzyme activity was determined using L-arabinose/D-galactose assay kit (Megazymes) except for synthetic substrates.

^b^No detectable activity was measured under the normal assay conditions.
